# Prevalence of pre-cancerous colon lesions in referred patients under patronage of a local relief foundation in Guilan province

**DOI:** 10.25122/jml-2018-0074

**Published:** 2019

**Authors:** Fariborz Mansour-Ghanaei, Gharmohammad Varshi, Farahnaz Joukar, Mohammad Taghi Ashoobi, Javad Esmaeilpour, Alireza Gharibpoor, Arash Daryakar, Roya Mansour-Ghanaei, Heydar Ali Balou, Hamid Saeidi Saedi, Sara Mavaddati, Masood Sepehrimanesh

**Affiliations:** 1. Gastrointestinal and Liver Diseases Research Center, Guilan University of Medical Sciences, Rasht, Iran; 2. GI Cancer Screening and Prevention Research Center, Guilan University of Medical Sciences, Rasht, Iran; 3. Caspian Digestive Disease Research Center, Guilan University of Medical Sciences, Rasht, Iran; 4. Università degli Studi di Bari Aldo Moro, Bari, Italy

**Keywords:** colorectal cancer, diverticulum, histopathology, neoplastic polyp

## Abstract

Colon cancer is the most commonly diagnosed gastrointestinal cancers in developed countries with varied incidence and the onset age of disease worldwide. Overall, 161 participants who were under patronage of a local relief foundation and referred to the endoscopy ward of Razi Hospital affiliated to the Guilan University of Medical Sciences. These patients have been aged more than 50 or more than 40 years with history of colorectal cancer in their first-degree family were enrolled from March 2016–March 2017. Demographic information were collected. Colonoscopy was performed and histopathological evaluation of observed lesions and polyps was done. Most of participants were female (113 individuals, 70.2%) and aged 50–60 years (83 individuals, 51.6%). Seventy-four (46%) had certain lesions. Most of colonoscopy findings were observed in the ascending colon in which depressed polyps and diverticulum were most frequent. However, rectum showed the most histological findings. All polyps of descending and ascending colons were neoplastic, while most of rectal polyps were non-neoplastic. Male patients, who were aged more than 60 years and smokers had significant higher percentage of both lesions and polyps in their colon (p<0.05). Moreover, significant positive association was detected between exposure to harmful industries and having polyps (p=0.01). We found male gender, higher age, smoking, and exposure to harmful industries as important risk factors for having colorectal lesions, which must be confirmed in further studies.

## Introduction

Colon cancer is one of the most important health issues and the most commonly diagnosed gastrointestinal (GI) cancers in developed countries [1]. It is estimated that normal persons have a 5–6% chance of developing colon cancer during their lifetime [2]. Colorectal cancer (CRC) is a common and fatal disease, which is the third and fourth most common cancers in women and men, respectively. CRC is the third most common cause of death in the world [3]. There is a significant difference in its distribution worldwide. More than 145,000 new cases of colon cancer are diagnosed every year in the United States, including a slightly more than 106,000 cases of colon cancer and other cases of rectal cancer. About 50,000 Americans die annually due to CRC, accounting for 9% of deaths from cancer in the country [4, 5].

Epidemiological features such as the incidence and the onset age of disease varied worldwide. Its annual prevalence in North America and Europe is approximately 30–50 per 100,000 while its prevalence in the Middle East is 3–7 per 100,000 [3]. Also, its prevalence is almost equal among men and women [5]. About 70–80% of CRC cases are sporadic and another 20–30% of patients have a history of disease in their first-degree relatives [6].

In Iran, GI cancers are the most common cancer among men and after breast cancer, the second most common cancer among women [7]. Another notable point is that the prevalence of colon cancer in men is higher among women, and more interesting is that the incidence of this cancer in young people in Iran is more than expected. Among the Asian countries, Iran has a high incidence of colon cancer that these findings may be due to Westernization of the diet, a change in the pattern of Iranian food consumption or an increase in smoking [8]. According to the little information and studies on epidemiological characteristics of CRC in Iran and Guilan province [9–11], we aimed to investigate the frequency of pre-cancerous colon lesions in patients under patronage of a local relief foundation in Guilan province as vulnerable population.

## Materials and Methods

### Patients

In a cross-sectional study from March 2016 to March 2017, all participants who were under patronage of a local relief foundation, referred to the endoscopy ward of Razi Hospital affiliated to the Guilan University of Medical Sciences with age more than 50 years or more than 40 years with history of CRC in their first-degree family were enrolled. All participants referred systematically by the Head of the Department of Health and Social Insurance of this local relief foundation from the cities over the Guilan province. Participants with history of colectomy and IBD were excluded. All included participants provided written informed consent and the aims of the study were described for all of them. The study protocol was approved by the Ethics Committee of the Gastrointestinal and Liver Diseases Research Center, Guilan University of Medical Sciences, and written informed consent (per the Helsinki declaration) was obtained from each participant.

### Data Collection

Demographic information about age, gender, height, weight, alcohol consumption, smoking, dietary regimen, physical activity and exposure to harmful industry were obtained from all patients using face-to-face questionnaire. Body mass index (BMI) was calculated which is expressed as kg/m^2^.

### Pre-procedure preparation colonoscopy and tissue sampling

All participants were prepared based on previous reported procedure [11] which started a day before the colonoscopy. Participant underwent endoscopy by an expert GI man. Conscious sedation using 1–2 mg Midazolam and 25 mg Petedin intravenous was used for all participants. Olympus GIF-CF140 video scopes was used following standard high-level liquid disinfection with Cidex 2.5% (Johnson & Johnson, USA) after cleaning and washing. Tissue sampling were done during colonoscopy from all observed lesions including polyps, ulcers, erosions, vascular lesions, mass and nodules. An expert pathologist evaluated all tissue samples after sectioning and staining with hematoxylin and eosin under light microscope.

### Statistical Analysis

SPSS version 20 was used for statistical analysis. Chi-square test for evaluation of any associations and logistic regression for estimation of odds ratio (OR) were used. p<0.05 was considered as significant difference. Figures were drawn using GrapPad Prism 7.0.

## Results

Totally 161 participants were included. Among them 152 one were aged higher than 50 years and other 9 persons had age lower than 50 years but had a history of CRC in their first-degree family. Most of participants were female (113 individuals, 70.2%), aged 50–60 years (83 individuals, 51.6%), married (155 persons, 96.3%), illiterate (89 participants, 55.3%), without smoking (133 persons, 82.6%) or alcohol consumption (160 participants, 99.4%). Most of our patients were housekeepers (105 ones, 65.2%) while farmers (32 persons, 19.9%), workers (13 individuals, 8.1%) and self-employed (11 participants, 6.8%) were in the next steps.

**Figure 1:**
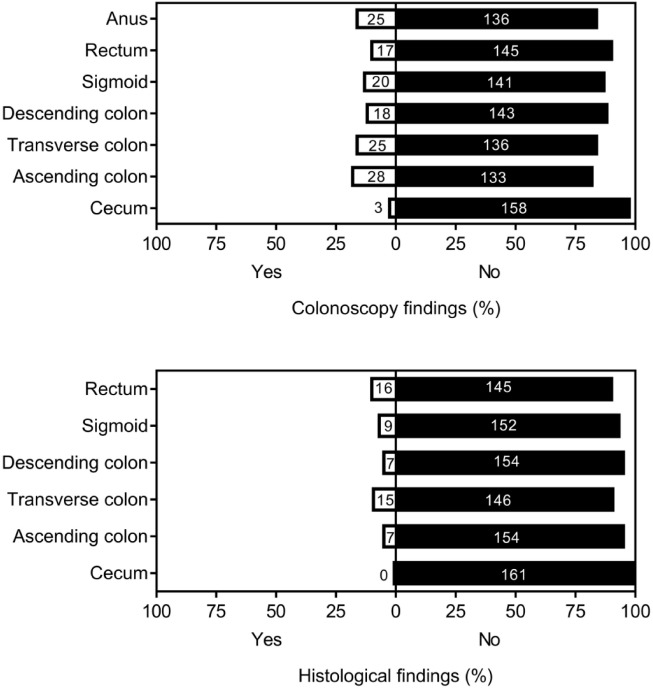
Distribution of colonoscopy and histological findings through the large intestine.

Among enrolled participants, 74 (46%) had certain lesions and other 87 persons (54%) showed no lesions in colonoscopy. Frequency and percentage of colonoscopy and histological findings based on different parts of large intestine are shown in Figure 1 and Table 1. Most of colonoscopy findings were observed in the ascending colon in which depressed polyps and diverticulum were most frequent. However rectum showed the most histological findings.

**Table 1:** Specific colonoscopy findings through the large intestine

	Anatomical site							
Colon								
**Lesions**	Cecum	Ascending	Transverse	Descending	Sigmoid	Rectum	Anal canal	Total
**Pin worm**	1	1	1	1	1	1	1	7 (5.43)
**Skin tag**	–	–	–	–	–	–	1	1 (0.77)
**Hemorrhoid**	–	–	–	–	–	–	23	23 (17.83)
**Erosion**	–	–	–	–	–	2	–	2 (1.55)
**Nodule**	–	1	–	–	–	2	–	3 (2.33)
**Mass**	–		–	–	–	3	–	3 (2.33)
**Ulcer**	–	1	1	1	2	1	–	6 (4.65)
**Polyp**								
Small sessile*	–	3	10	4	3	8	–	28 (21.70)
**Non-small sessile**	1	2	–	–	2	–	–	5 (3.88)
**Pediculated**	–	2	3	2	2	–	–	9 (6.98)
**Ectasia**	–	–	–	–	1	–	–	1 (0.77)
**Diverticula**	2	10	10	10	9	–	–	41 (31.78)
**Total**	4 (3.10)	20 (15.50)	25 (19.38)	18 (13.95)	20 (15.50)	17 (13.18)	25 (19.38)	129 (100)

* Small sessile polyp <5 mm; Non-small sessile polyp ≥5 mm.

**Figure 2:**
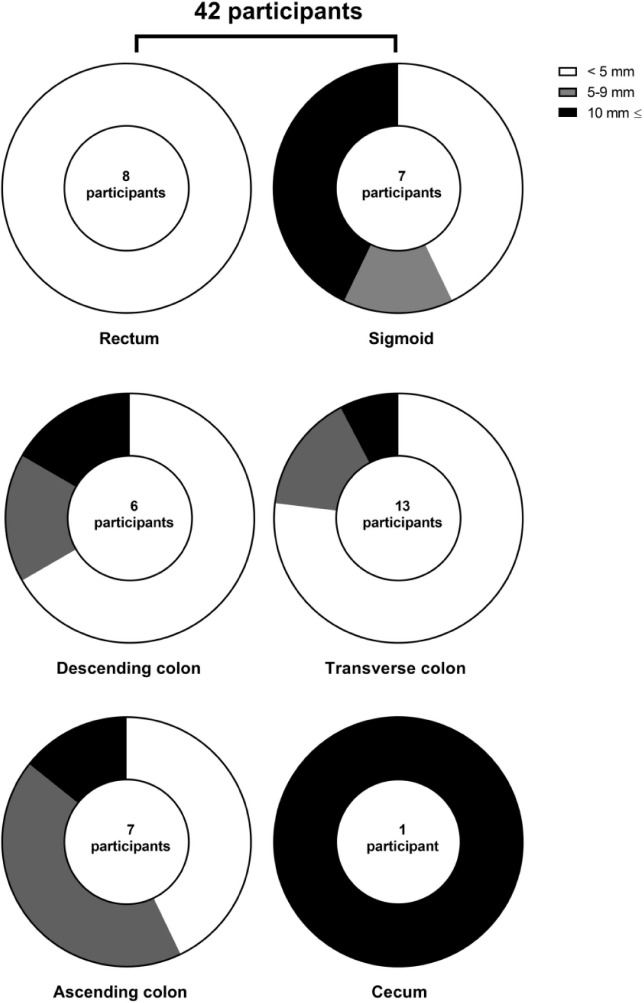
Lesions’ size in different anatomical parts of large intestine.

Distribution of lesions’ size in different anatomical parts of large intestine is presented in Figure 2. All lesions in rectum were smaller than 5 mm while the only lesion in the cecum was bigger than 10 mm.

Among suspicious specimens in colonoscopy, which sent for more histopathological evaluation, all polyps of descending and ascending colons were neoplastic (adenomatous), while most of rectal polyps were non-neoplastic. Most of neoplastic polyps were adenomatous type which seen mostly in the sigmoid and transverse colon. Just two hyperplastic polyps were detected which been in rectum and sigmoid (Table 2).

**Table 2:** Frequency (percentage) of neoplastic and non-neoplastic polyps in different anatomical parts of large intestine. Frequency of other lesion in each part also presented

Anatomical site	Type of polyp			Other lesions
	Adenomatous	Hyperplastic	Non-neoplastic	
**Rectum**	3 (25)	1 (8.3)	8 (66.7)	Two adenocarcinoma, 1 non-specific colitis, 1 IBD
**Sigmoid**	7 (77.8)	1 (11.1)	1 (11.1)	One IBD
**Descending colon**	4 (100)	0 (0)	0 (0)	One non-specific colitis, 1 IBD
**Transverse colon**	7 (58.3)	0 (0)	5 (41.7)	Two non-specific colitis, 1 IBD
**Ascending colon**	3 (100)	0 (0)	0 (0)	One non-specific colitis, 1 IBD
**Cecum**	0 (0)	0 (0)	0 (0)	One submucosal lipoma

Male patients, who are aged more than 60 years and smokers had significant higher percentage of both lesions and polyps in their colon. Moreover, significant positive association was detected between exposure to harmful industries and having polyps (p=0.01, Table 3).

**Table 3:** Associations of demographic data and socioeconomic state with lesions and polyps

Variables	**Lesions**			**Polyps**		
	Yes	No	p-Value	Yes	No	p-Value
**Age (years)**			0.05			0.01
<50	8 (47.1)	9 (52.9)		4 (23.5)	13 (76.5)	
50–60	31 (37.3)	52 (62.7)		14 (16.8)	69 (83.2)	
>60	35 (57.4)	26 (42.6)		23 (37.7)	38 (62.3)	
**Gender**			0.001			0.001
Male	32 (66.7)	16 (33.3)		21 (43.7)	27 (56.3)	
Female	42 (37.2)	71 (62.8)		20 (17.6)	93 (82.4)	
**BMI (kg/m^2^)**			0.8			0.2
<25	28 (49.1)	29 (50.8)		18 (31.5)	39 (68.5)	
25–30	25 (43.8)	32 (56.2)		15 (26.3)	42 (73.7)	
>30	21 (44.7)	26 (55.3)		8 (17)	39 (83)	
**Marital status**			0.2			0.05
Single	2 (33.3)	4 (66.7)		1 (16.7)	5 (83.3)	
Married	44 (52.4)	40 (47.6)		28 (33.3)	56 (66.7)	
Divorced	28 (39.4)	43 (60.6)		12 (16.9)	59 (83.1)	
**Education**			0.9			0.08
Illiterate	40 (44.9)	49 (55.1)		20 (22.5)	69 (77.5)	
Before diploma	30 (46.8)	34 (53.2)		18 (28.1)	46 (71.9)	
Diploma	3 (50)	3 (50)		1 (16.7)	5 (83.3)	
Academic	1 (50)	1 (50)		2 (100)	0 (0)	
**Exposure to harmful industries**			0.3			0.01
Yes	14 (51.8)	13 (48.2)		12 (44.4)	15 (55.6)	
No	60 (44.8)	74 (55.2)		29 (21.6)	105 (78.4)	
**Physical activity**			0.4			0.1
Yes	20 (43.5)	26 (56.5)		31 (67.4)	15 (32.6)	
No	54 (46.9)	61 (53.1)		89 (77.4)	26 (26.6)	
**Smoking**			0.003			0.005
Yes	20 (71.4)	8 (28.6)		13 (46.4)	15 (53.6)	
No	54 (40.6)	79 (59.4)		28 (21.1)	105 (78.9)	
**Alcohol consumption**			0.460			0.255
Yes	1 (100)	0 (0)		1 (100)	0 (0)	
No	73 (45.6)	87 (54.4)		40 (25)	120 (75)	
**Familial history of cancers**			0.5			0.5
Grade 1	22 (44.9)	27 (55.1)		11 (22.4)	38 (77.6)	
Grade 2	14 (46.7)	16 (53.3)		6 (20)	24 (80)	
Grade 3	7 (63.6)	4 (36.4)		3 (27.3)	8 (72.7)	

**Figure 3:**
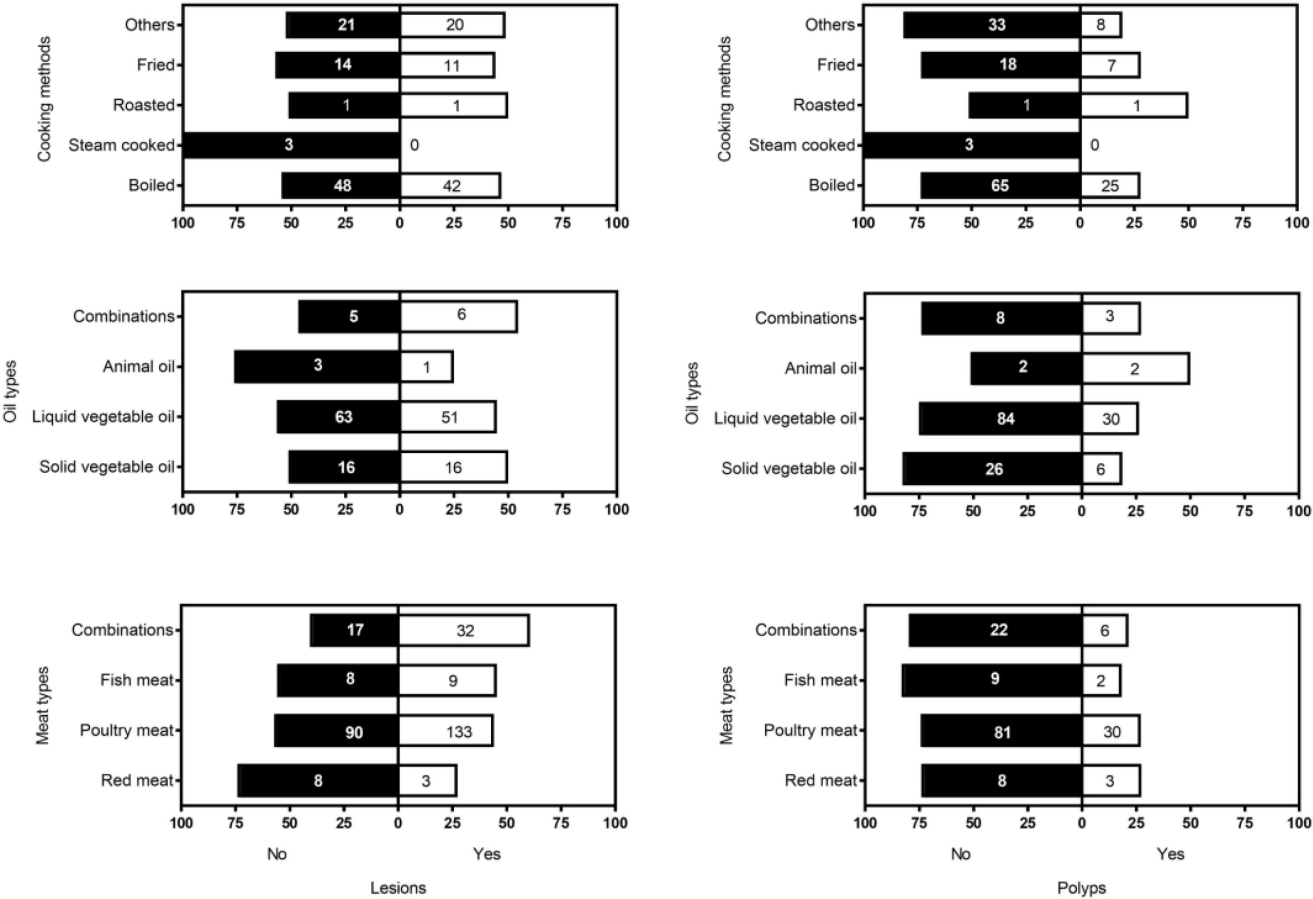
Frequency and percentage of lesions (left) and polyps (right) based on some dietary habitations include cooking methods, oil types and meat types plus physical activity.

Associations between dietary habitations with colonoscopy lesions and polyps are presented in Figure 3. No significant association detected between dietary habitations with neither lesions nor polyps (p>0.05).

Finally, regression model study revealed that just male sex, higher age for colonoscopy lesions and exposure to harmful industries for polyps were the predictive risk factors in this specific population (Table 4).

**Table 4:** Regression model of certain demographic variables for intestinal metaplasia and H. pylori infection

Variables	Lesions	Polyps
**Sex**		
**Male**	3.3 (1.6–6.8)*	0.2 (0.1–0.5)*
**Female**	Ref.	Ref.
**Age (years)**		
**<50**	1.5 (0.5–4.4)	0.5 (0.1–1.7)
**50–60**	2.2 (1.1–4.4)*	0.3 (0.1–0.7)*
**>60**	Ref.	Ref.
**BMI (kg/m^2^)**		
**<25**		2.2 (0.8–5.7)
**25–30**		1.7 (0.6–4.5)
**>30**		Ref.
**Exposure to harmful industries**		
**Yes**		Ref.
**No**		0.3 (0.1–0.8)*
**Physical activity**		
**Yes**		0.3 (0.2–1.2)
**No**		Ref.

* p<0.05.

## Discussion

Colon cancers is one of the major health issues and the most commonly diagnosed GI cancers in developed countries [12]. Identification of at risk people is possible based on genetic and other screening tests. Since these screening programs have been implemented, there has been considerable success in early detection, prevention, and treatment of colon cancer. In developed countries, screening programs for early diagnosis and prevention of colon cancer are considered as anti-cancer strategies and one of the approved cancer control programs. In the present study, we monitored the pre-cancerous lesions in the colon of individuals covered by a local relief committee to diagnose their potential disease in the early stages.

We found most lesions in the rectum, which is similar to previous reports. For instance, Abdollahi and Faizollah reported that the most prevalent site for colorectal carcinoma was rectum followed by sigmoid colon [13]. We diagnosed two cases of adenocarcinoma type of CRC also in the rectum, which is similar to previous report by Adibfar et al. They found that more than 91% of CRCs were adenocarcinoma type [14]. It has been reported that most cases of CRC develop after long-term alteration of adenomatous polyps. Therefore, a quick diagnosis and treatment of polyps is the most effective way to prevent CRC [8]. We detected most of polyps only for males who are more than 60 years of age. Previous studies confirmed that the incidence of polyps were increased with increment of age and people with age of 70–75 years had polyps two times more than individuals of age 40–49 years [15, 16]. Agah and coworkers also reported that the most detected polyps (27.5%) was detected in the age range of 60–70 years [17]. Furthermore, Joukar et al. reported that polyps were mostly seen in patients with age more than 40 years while most adenocarcinomas were seen in patients aged more than 60 years [11]. Almost all studies found that the colon cancer is most prevalent in higher age male groups and these are unrelated to the population or geographic regions [18–24]. Furthermore, about 90% of new cases of CRC are diagnosed in persons aged more than 50 years [25].

We found no significant association between having lesions or polyps with familial history of cancer. This finding is in contrast with some of those reported previously. Fatemi and coworkers compared the colonoscopy findings of 90 first-degree relatives of CRC patients with 94 age and sex matched, average risk control patients with no family history of CRC. Finally, they reported that the prevalence of pre-cancerous lesions in first-degree relatives of patients diagnosed with CRC is significantly higher than in the average risk population [26]. Fuchs and colleagues also reported that a family history of CRC is associated with an increased risk of this disease especially in younger patients [27]. Moreover, Ahsan et al. confirmed that first-degree relatives of patients with newly diagnosed adenomas are more at risk for CRC [28]. On the other hand, it has been reported that the risk of CRCs is increased by smoking, alcohol intake and increased BMI [29]; but we found just significant effects for smoking in our patients. This may be due to lack of self-reported alcohol consumption and slow sample size.

Certain animal model studies described the associations between red meat consumption and increase risk of colon cancer which systematically analyzed by Turner and Lloyd [30]. They expressed the causes of cancer in association to heme iron and nitroso-compounds [31–34] or cooking effects [35]. However, we could not find such association between the type of consumed meat, oil, and cooking method with colon cancer, which may be due to low sample size of this study.

Fang et al. reported that industries with exposure to wood dusts and ammonia could increase occupational risk of colon cancer [36]. However, in the evaluation of 15 million people in five Nordic countries, Pukkala and co-workers found that the colon cancer showed the small relative variation in incidence between occupational categories [37]. In addition, it has been reported that professional and other white collar workers, chemical processors and textile workers were at high risk of colon cancer [38]. Although we found significant association between exposures to harmful industries and having polyp as pre-cancerous lesion, but the type of industries were not addressed in our study and must be considered in further studies.

## Limitations

Despite of our findings, this study is a minor limitation including lack of data about the types of harmful exposure. However, we will invite persons with exposure to harmful industries and will ask a complete list of questions about the nature of exposure in the future study.

## Conclusions

In summary, we found male gender, higher age, smoking and exposure to harmful industries as risk factor for colon polyps but just male gender and higher age had significant associations with colorectal polyps. Performing further studies with higher sample size and mostly focused on the occupational hazards are highly recommended.

## Acknowledgements

We would like to thank Endoscopy and Colonoscopy ward staff of Razi Hospital who assisted us in this study.

## Conflict of Interest

The authors confirm that there are no conflicts of interest.
